# Gastric Aspiration and Its Role in Airway Inflammation

**DOI:** 10.2174/1874306401812010083

**Published:** 2018-12-26

**Authors:** EB Hunt, A Sullivan, J. Galvin, J. MacSharry, DM Murphy

**Affiliations:** 1 The Department of Respiratory Medicine, Cork University Hospital, Cork, Ireland; 2 The Health Research Board Clinical Research Facility, University College Cork, Cork, Ireland; 3 The APC Microbiome Institute, Schools of Medicine and Microbiology, University College Cork, Ireland

## Gastric Aspiration and Its Role in Airway Inflammation

The Open Rheumatology Journal, **2018**, 12: 1-10

The type of Article has been revised as follows:

**Review Article**

The type of article originally provided was:

**Research Article**

The revised headings of declarations are mentioned below:

**CONSENT FOR PUBLICATION:**

Not applicable.

The original headings of declarations provided were:

**ETHICS APPROVAL AND CONSENT TO PARTICIPATE**

Not applicable.

**HUMAN AND ANIMAL RIGHTS**

No Animals/Humans were used for studies that are base of this research.

